# Preoperative functional connectivity and postoperative neuropathic pain in degenerative cervical myelopathy: An exploratory postoperative follow-up study using resting-state functional MRI

**DOI:** 10.1016/j.bas.2026.106661

**Published:** 2026-08-29

**Authors:** Takane Nakagawa, Fumihiko Eto, Tomoyuki Asada, Kento Inomata, Yosuke Ogata, Kotaro Sakashita, Takahiro Sunami, Tomoaki Shimizu, Shun Okuwaki, Hisanori Gamada, Kousei Miura, Hiroshi Noguchi, Toru Funayama, Masao Koda, Masashi Yamazaki, Hiroshi Takahashi

**Affiliations:** a Department of Orthopaedic Surgery, Institute of Medicine, University of Tsukuba, 1-1-1, Tennodai, Tsukuba City, Ibaraki, 305-8575, Japan; b Department of Orthopaedic Surgery, NHO Mito Medical Center, 280, Sakuranosato, ibarakimachi, Ibaraki, 311-3193, Japan; c Hospital for Special Surgery, 535 E 70th St, New York, NY, USA; d Department of Orthopaedic Surgery, Kenpoku Medical Center Takahagi Kyodo Hospital, 1006-9, Kamitezunaagehocho, Takahagi, Ibaraki, 318-0004, Japan; e Department of Orthopaedic Surgery, Moriya Daiichi General Hospital, 1-17, Matsumaedai, Moriya, Ibaraki, 302-0102, Japan; f Department of Orthopaedic Surgery, Tsukuba Medical Center Hospital, 1-3-1, Amakubo, Tsukuba, Ibaraki, 305-8558, Japan; g Department of Orthopaedic Surgery, Ushiku Aiwa General Hospital, 896, Inokocho, Ushiku, Ibaraki, 300-1296, Japan; h Department of Orthopaedic Surgery, Ichihara Hospital, 3681, Ozone, Tsukuba, Ibaraki, 300-3253, Japan

**Keywords:** Degenerative cervical myelopathy, Neuropathic pain, Functional connectivity, Rs-fMRI

## Abstract

**Introduction:**

Neuropathic pain (NP) may persist after decompression surgery for degenerative cervical myelopathy (DCM). Although clinical factors associated with postoperative NP have been reported, neuroimaging correlates based on functional connectivity (FC) remain poorly understood.

**Research question:**

Are specific preoperative resting-state FC patterns associated with postoperative NP status in patients with DCM?

**Material and methods:**

In this retrospective observational study using prospectively collected data, we analyzed 34 patients who underwent cervical decompression surgery for DCM between 2020 and 2024. NP was assessed using the Spine painDETECT Questionnaire (SPDQ) preoperatively and 6 months postoperatively. Patients were classified into NP improvement group (n = 7) and non-improvement group (n = 27). Preoperative resting-state functional MRI data underwent whole-brain ROI-to-ROI FC analysis using the CONN toolbox. Between-group FC differences, correlations with patient-reported outcomes, and factors associated with postoperative SPDQ scores were evaluated.

**Results:**

Two preoperative FC networks showed significant between-group differences. FC1, between the right middle frontal gyrus and right thalamus, was higher in the NP improvement group (p-FDR = 0.034), whereas FC2, between the left temporal fusiform cortex and left occipital fusiform gyrus, was higher in the NP non-improvement group (p-FDR = 0.024). FC2 showed moderate correlations with changes in pain intensity, quality of life, and upper-limb function. Multivariable analysis identified FC1 as independently associated with postoperative SPDQ scores (β = −24.05, p = 0.001).

**Discussion and conclusion:**

Distinct preoperative FC patterns were associated with postoperative NP status. Preoperative FC may provide exploratory neuroimaging correlates of postoperative NP, although further validation is warranted.

## Highlights

• Preoperative rs-fMRI identified FC patterns associated with postoperative NP status.
• FC1 (right middle frontal gyrus–thalamus) was higher in the NP improvement group.
• FC2 (temporal fusiform–occipital fusiform) was higher in the non-improvement group.
• FC1 was independently associated with postoperative SPDQ scores.
• Preoperative FC patterns may represent candidate biomarkers for postoperative NP.

## 1 Introduction

Degenerative cervical myelopathy (DCM) is a spinal disorder that causes pain, numbness, motor dysfunction, and impaired quality of life (QOL). Surgical decompression is the standard of care for progressive or moderate-to-severe disease; however, spinal cord-related neuropathic pain (NP) may persist after surgery. NP, defined as pain caused by a lesion in the somatosensory nervous system, is a prominent contributor to residual postoperative symptom burden (Finnerup et al., 2021). It is present in approximately 75% of patients with DCM (Yamashita et al., 2014), and poor postoperative improvement has been reported in 66.3% of patients with spinal cord-related NP (Nakajima et al., 2022). A recent multicenter prospective study of 816 patients with DCM reported preoperative neuropathic pain symptoms in 88.5% of patients, with paresthesia/dysesthesia remaining the most persistent symptom at 2 years postoperatively (Nagoshi et al., 2026). Stronger preoperative pain has also been associated with postoperative NP (Nakajima et al., 2022). These observations emphasize the need for objective preoperative markers that can identify patients at risk for refractory NP beyond patient-reported screening tools.

Resting-state functional magnetic resonance imaging (rs-fMRI) estimates functional connectivity (FC) from temporal correlations in spontaneous blood-oxygenation-level-dependent (BOLD) signal fluctuations (Ogawa et al., 1990; Greicius et al., 2003; Raichle and Snyder, 2007; Logothetis et al., 2001; Lee et al., 2010). Because the BOLD signal is an indirect hemodynamic measure rather than a direct measure of neuronal activity or cerebral oxygen metabolism, FC estimates may be influenced by regional differences in neurovascular coupling (Epp et al., 2026). Rs-fMRI has been widely used to assess psychiatric disorders, neurological diseases, and DCM (Eto et al., 2022; Greicius et al., 2004; Ishikawa et al., 2022; Mi et al., 2017). The rs-fMRI studies on NP, such as trigeminal neuralgia, have consistently highlighted the association between FC abnormalities and the thalamocortical and insula-anterior cingulate network (Chen et al., 2025; Nardoni et al., 2025; Zhang et al., 2021). In contrast, although studies on DCM have demonstrated that postoperative changes in FC are associated with motor function recovery, few studies have investigated the association between FC and NP status after DCM surgery (Eto et al., 2022; Bhagavatula et al., 2016; Takenaka et al., 2020; Khan et al., 2024).

In this context, preoperative FC assessment using rs-fMRI could provide exploratory neuroimaging correlates of postoperative NP outcomes in patients with DCM. Therefore, we hypothesized that abnormalities in specific preoperative FC patterns in patients with DCM were associated with NP status and postoperative clinical outcomes. Accordingly, we performed an exploratory comprehensive FC analysis using rs-fMRI to identify potential factors for postoperative non-NP status.

## 2 Methods

### 2.1 Study design and patient selection

This was a retrospective observational study using prospectively collected clinical and imaging data from patients who underwent cervical decompression surgery for DCM at our institution between June 2020 and July 2024. Clinical outcomes and rs-fMRI data were prospectively collected at predefined preoperative and 6-month postoperative time points, whereas the present research question and analyses were defined retrospectively. DCM was diagnosed by board-certified spine surgeons based on neurological examinations, clinical symptoms, and preoperative cervical MRI showing spinal cord compression. To minimize potential confounding from cervical radiculopathy, patients with concomitant symptomatic cervical foraminal stenosis considered responsible for upper-extremity radicular pain, based on neurological examination and preoperative cervical MRI, were not included in the source population. Surgical indications were determined by the attending board-certified surgeons during an internal case conference.

The study was conducted in accordance with the principles of the Declaration of Helsinki. Informed consent was obtained from all the participants, and the study protocol was approved by the institutional ethics committee. This study was reported in accordance with the STROBE (Strengthening the Reporting of Observational Studies in Epidemiology) guidelines.

**Fig. 1:**
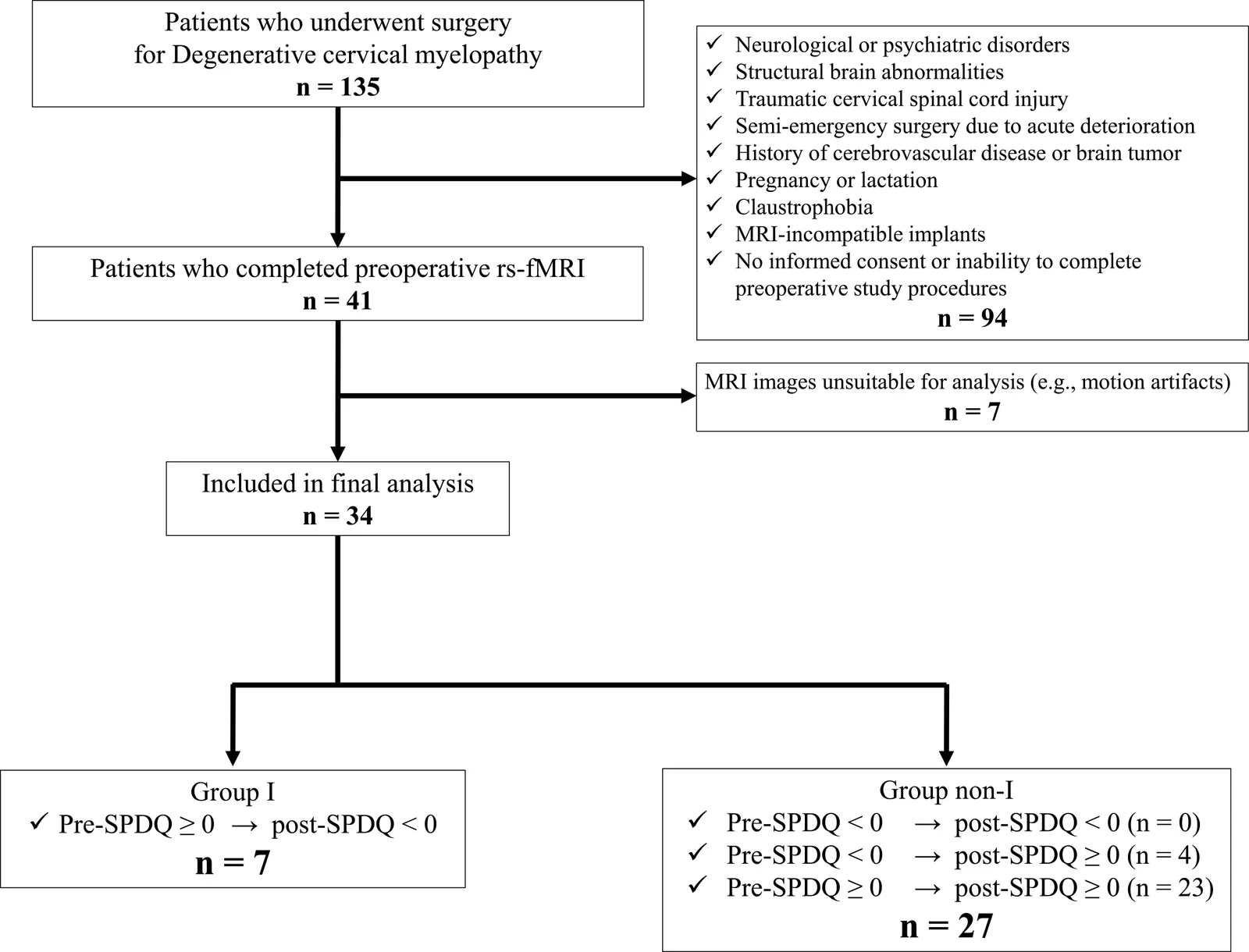
Patient selection flow diagram. Flow diagram showing patient screening, exclusion, final inclusion, and classification into the neuropathic pain improvement and non-improvement groups. Of 135 patients in the source population, 41 completed preoperative rs-fMRI, and 34 were included in the final analysis after imaging quality control. Abbreviations: MRI, magnetic resonance imaging; SPDQ, Spine painDETECT Questionnaire.

The inclusion criteria were patients who underwent cervical spine surgery for DCM at our institution during the study period and provided informed consent to participate. The exclusion criteria were as follows: (1) neurological or psychiatric disorders, (2) structural brain abnormalities, (3) traumatic cervical spinal cord injury, (4) semi-emergency surgery due to acute deterioration, (5) history of cerebrovascular disease or brain tumor, (6) pregnancy or lactation, (7) claustrophobia, and (8) MRI-incompatible implants. Of the 135 patients in the source population, 41 provided informed consent and completed preoperative rs-fMRI. Enrollment was limited by the need to complete the research procedures before surgery, which was not always feasible, particularly in patients requiring expedited surgery. After excluding seven patients because of inadequate rs-fMRI data quality, 34 patients were included in the final analysis (Fig. 1).

Although the sample size (n = 34) was modest, it was comparable to that of previous rs-fMRI studies of DCM (Eto et al., 2022; Chen et al., 2018; Wu et al., 2024). Given the technical and logistical constraints of advanced neuroimaging, this study was intended to be an exploratory hypothesis-generating analysis.

### 2.2 Demographic data and clinical outcomes

Demographic data included age, sex, DCM pathology (ossification of the posterior longitudinal ligament, cervical spondylosis, or cervical disc herniation), American Society of Anesthesiologists Physical Status (ASA-PS), preoperative NP-related medication (pregabalin or mirogabalin), symptom duration, surgical approach (anterior vs. posterior), operated levels, operation time (minutes), and intraoperative blood loss (g). Demographic and surgical data were retrospectively extracted from electronic medical records, whereas clinical outcome and imaging data were prospectively collected at the predefined preoperative and 6-month postoperative time points. Symptom duration was collected based on the patient's interview and dichotomized using a 12-month cutoff based on a previous study (Asuzu et al., 2022).

Clinical outcomes included patient-reported outcome measures (PROMs): the Numerical Rating Scale (NRS; a measure of pain intensity), the EuroQol 5 Dimensions, 5-Level version (EQ-5D; a generic health-related QOL instrument), Neck Disability Index (NDI; a questionnaire assessing disability and daily impact from neck pain), Short Form Health Survey-8 (SF-8; a brief overall health survey), the Japanese Orthopaedic Association score (JOA score), and the Japanese Orthopaedic Association Cervical Myelopathy Evaluation Questionnaire (JOACMEQ; a disease-specific patient-reported outcome measure for DCM). These clinical outcomes were assessed preoperatively and at 6 months postoperatively.

### 2.3 NP assessment

We assessed NP status using the Spine painDETECT Questionnaire (SPDQ). A score of ≥ 0 was defined as the presence of NP, and a score of < 0 was defined as the absence of NP (Nikaido et al., 2018). NP change was evaluated in patients who completed SPDQ assessments both preoperatively and 6 months postoperatively. Based on these assessments, the patients were classified into two groups:

• NP improvement group (Group I, n = 7), defined as patients with SPDQ ≥ 0 preoperatively and SPDQ < 0 6 months postoperatively; and
• NP non-improvement group (Group non-I, n = 27), which included patients who showed worsening NP (SPDQ < 0 to ≥ 0, n = 4) or persistent NP (SPDQ ≥ 0 throughout, n = 23).

The terms “improvement” and “non-improvement” referred only to categorical transitions in SPDQ-defined NP status and did not indicate the presence or absence of overall clinical benefit from surgery.

### 2.4 Rs-fMRI acquisition and preprocessing

All MRI scans were performed using a Philips 3.0 T Achieva dStream scanner (Philips Healthcare, Cleveland, OH, USA). The patients were placed in the supine position and instructed to fixate on a central cross displayed on the screen. Functional images were acquired using a blood oxygenation level-dependent (BOLD) gradient-echo planar pulse sequence (repetition time [TR] = 2500 ms, echo time [TE] = 30 ms, slice thickness = 3.2 mm, field of view [FOV] = 212 × 212 mm, flip angle = 80°, 40 slices, 240 vol) over 10 min. Additionally, a three-dimensional isotropic magnetization-prepared rapid acquisition gradient-echo sequence (TR = 7.4 ms, TE = 3.5 ms, slice thickness = 1.2 mm, FOV = 256 × 240 mm, flip angle = 9°, 170 slices) was obtained for structural alignment.

Data preprocessing was performed using the CONN toolbox (version 22.a; http://www.nitrc.org/projects/conn) running on MATLAB R2017b (The MathWorks, Inc., Natick, MA, USA) within the Lin4Neuro environment (Ubuntu 18.04–based) (Whitfield-Gabrieli and Nieto-Castanon, 2012). The following preprocessing steps were applied to each patient's data: realignment, slice-timing correction, coregistration to structural images, spatial normalization to the Montreal Neurological Institute standard template (resampled to a voxel size of 2 × 2 × 2 mm^3^), and spatial smoothing using a 6-mm full-width at half-maximum Gaussian kernel. Noise components from the white matter, cerebrospinal fluid, and motion parameters were regressed out, followed by bandpass filtering at 0.008–0.09 Hz (Weissenbacher et al., 2009). Patients with excessive head motion were excluded if the number of valid volumes after correction and scrubbing was < 200 of the 240 total volumes. Only datasets with at least 200 valid volumes were included in the analyses (seven patients were excluded based on this criterion), following previous rs-fMRI quality control standards recommending exclusion when more than 15–20% of the volumes were affected by motion artifacts (Power et al., 2012).

### 2.5 Functional connectivity analysis

The FC analyses were performed using the CONN toolbox. We employed a seed-based ‘ROI-to-ROI approach,’ a method widely used in cervical myelopathy studies (Eto et al., 2022; Woodworth et al., 2018), in which each predefined region of interest (ROI) was treated as a seed and its mean BOLD time series was correlated with every other ROI. The ROI set comprised 132 regions from the Harvard–Oxford anatomical atlas and 32 network-derived ROIs from the CONN network atlas (covering the default mode, sensorimotor, and salience systems), yielding 164 ROIs in total and allowing for a comprehensive whole-brain analysis. The mean time series were extracted from each ROI, pairwise Pearson correlations were computed to form a 164×164 FC matrix, and the coefficients were Fisher's r-to-z-transformed before group-level analyses. FC was computed at baseline (preoperative) and 6 months postoperatively.

FC values were compared between the predefined groups based on the NP status (Group I and Group non-I) to identify specific connections that showed statistically significant group differences. To account for the increased risk of type I error due to multiple comparisons across numerous ROI pairs, statistical significance was set at *p* < 0.05, after applying a false discovery rate correction. The false discovery rate correction was applied across all ROI-to-ROI connections included in the whole-brain analysis, thereby controlling for multiple testing across the complete set of examined functional connectivity pairs.

### 2.6 Statistical analysis

Analyses were performed using the SPSS Statistics software (version 30.0.0.0; IBM Corp., USA). For clinical outcomes, the Shapiro–Wilk test was used for normality assessment. Continuous values with normal distributions were described as mean ± standard deviation, and non-normally distributed values were described as median. All differences were defined as postoperative minus preoperative scores, and each difference was denoted with the prefix Δ. Categorical values were presented as frequencies.

For group comparisons, the Student's t-test was applied when normality and homogeneity of variance were confirmed, and Welch's *t*-test was used otherwise. The Mann–Whitney *U* test was used for nonparametric comparisons.

Correlations were examined between preoperative FC of the right middle frontal gyrus–right thalamus network (FC1), the left temporal fusiform cortex–left occipital fusiform gyrus network (FC2), and ΔPROMs. As an exploratory analysis, we performed correlation analyses between ΔSPDQ and ΔPROMs. All correlations were computed using Spearman's rank correlation, which is appropriate for ordinal or non-normally distributed data common in spine research (Crawford et al., 2025). Correlation coefficients were interpreted as follows: values below 0.30 were considered mild, values between 0.30 and 0.70 were considered moderate, and values of 0.70 or higher were considered strong (Fujimori et al., 2024).

To further explore the association between FC and postoperative SPDQ scores, multivariate linear regression analysis was performed with independent variables such as age, sex, preoperative NRS, FC1, and FC2 based on univariate analysis and clinical relevance. The effect of each factor was assessed by coefficient and 95% confidence intervals (β [95% CIs]). The overall model fit was assessed using the R^2^ values. Statistical significance was set at *p* < 0.05.

## 3 Results

### 3.1 Preoperative clinical characteristics

Among the 34 patients included in the analysis, there were no significant differences between Groups I and non-I in terms of age, sex, diagnosis, ASA-PS, preoperative NP-related medications, symptom duration, and surgical approach. The Group non-I was more likely to have male patients, although this difference was not statistically significant (81.5% vs. 57.1%, *p* = 0.32). Symptom duration longer than 12 months was likely to be more frequent in Group I patients, but the difference was not statistically significant (85.7% vs. 40.7%, *p* = 0.09) (Table 1).

**Table 1:** Preoperative clinical characteristics and demographic data.

Variable	Group I (n = 7)	Group non-I (n = 27)	*p*-value
Age, years	68.9 ± 15.5	63.7 ± 10.6	0.43^a^
Sex, male	4 (57.1%)	22 (81.5%)	0.32^b^
Diagnosis			
Ossification of Posterior Longitudinal Ligament	4	8	0.21^b^
Cervical Spondylosis	3	18	0.39^b^
Cervical Disc Herniation	0	1	1.00^b^
ASA-PS			0.80^b^
1	0	1	
2	3	14	
3	4	11	
4	0	1	
Preoperative NP-related medication	2 (28.6%)	11 (40.7%)	0.68^b^
Pregabalin	2	6	1.00^b^
Mirogabalin	0	5	0.56^b^
Symptom duration (≥12 months)	6 (85.7%)	11 (40.7%)	0.09^b^
Surgical approach, posterior (%)	5 (71.4%)	15 (55.6%)	0.67^b^
Operated levels (n)	3.3 ± 1.3	3.8 ± 1.8	0.63^c^
Operation time (minutes)	185.3 ± 143.9	235.3 ± 98.2	0.06^c^
Blood loss (g)	86.0 ± 80.8	172.3 ± 266.3	0.63^c^

Continuous variables are shown as mean ± SD or median [IQR], as appropriate. Statistical significance was set at *p* < 0.05.

Abbreviations: ASA-PS, American Society of Anesthesiologists Physical Status.

a Welch's *t*-test.

b Fisher's exact test.

c Mann–Whitney *U* test.

### 3.2 Preoperative FC values and PROMs

Preoperative ROI-to-ROI analysis identified two FC networks with significant between-group differences. The FC1 was significantly higher in Group I (*p* = 0.034), while the FC2 was significantly higher in Group non-I (*p* = 0.024). For preoperative PROMs, there were no significant differences between Group I and Group non-I in terms of all PROMs except NRS, which was significantly higher in Group non-I (6 [5-8] vs. 3 [2-5], *p* = 0.023) (Table 2).

**Table 2:** Preoperative PROMs and FC values.

Variable	Group I (n = 7)	Group non-I (n = 27)	*p*-value
**NRS**	**3 [2-5]**	**6 [5-8]**	**0.023**^a^
EQ-5D	0.67 ± 0.21	0.64 ± 0.2	0.75^b^
NDI	9.6 ± 6.2	11.8 ± 8.5	0.51^b^
SF-8			
PCS	34.0 ± 8.3	35.6 ± 8.5	0.65^c^
MCS	43.7 ± 10.2	45.4 ± 7.1	0.61^c^
JOA score	11.0 ± 2.81	10.7 ± 1.95	0.80^b^
JOACMEQ			
Cervical spine function	60.7 ± 33.4	65.2 ± 21.9	0.75^b^
Upper limb function	73.1 ± 21.1	71.9 ± 19.7	0.89^b^
Lower limb function	50.6 ± 29.6	57.0 ± 28.3	0.62^b^
Bladder function	73.3 ± 21.1	73.2 ± 23.2	1.00^b^
QOL	41.0 ± 20.3	43.6 ± 14.8	0.87^b^
**FC1 values**	**0.16 **±** 0.13**	**−0.09 **±** 0.13**	**0.034**^d^
**FC2 values**	**−0.03 **±** 0.13**	**0.19 **±** 0.13**	**0.024**^d^

Continuous variables are shown as mean ± SD or median [IQR], as appropriate. Statistical significance was set at *p* < 0.05.

Abbreviations: PROMs, Patient Reported Outcome Measures; NRS, Numerical Rating Scale; EQ-5D, EuroQol 5 Dimensions, 5-Level version (EQ-5D-5L); NDI, Neck Disability Index; SF-8, Short Form-8 Health Survey; PCS, Physical Component Summary; MCS, Mental Component Summary; JOA, Japanese Orthopaedic Association; JOACMEQ, JOA Cervical Myelopathy Evaluation Questionnaire; QOL, Quality of Life; IQR, Interquartile Range.

a Mann–Whitney *U* test.

b Welch's *t*-test.

c Student's t-test.

d p-values are false discovery rate-corrected for multiple comparisons.

### 3.3 Postoperative FC values and PROMs at 6 months

**Fig. 2:**
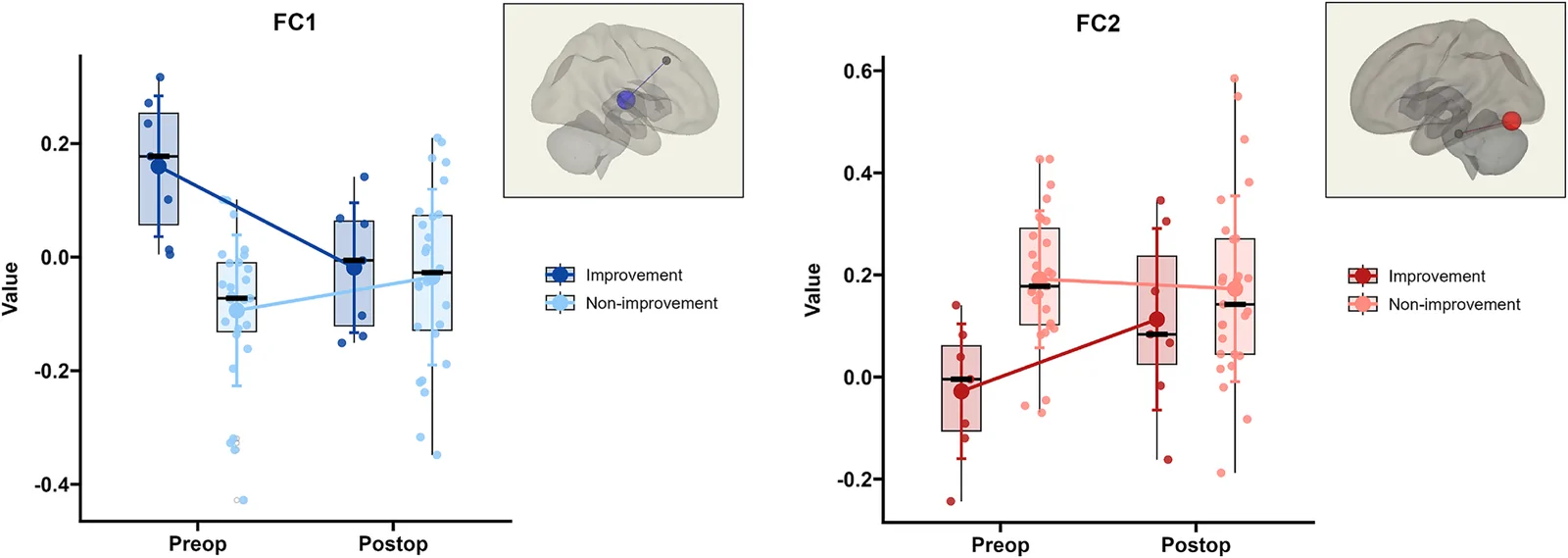
Preoperative and postoperative functional connectivity values in FC1 and FC2. Box-and-dot plots showing preoperative and 6-month postoperative functional connectivity values for FC1 and FC2 in the neuropathic pain improvement and non-improvement groups. Brain renderings (FC1: right lateral view; FC2: left anterolateral view) depict the corresponding networks. FC1 represents connectivity between the right middle frontal gyrus (large blue sphere) and the right thalamus (small gray sphere). FC2 represents connectivity between the left temporal fusiform cortex (small gray sphere) and the left occipital fusiform gyrus (large red sphere). Abbreviations: FC, functional connectivity.

Postoperatively, the FC1 and FC2 values were not significantly different between the two groups, and the preoperative to postoperative changes in the FC values are shown in Fig. 2. Additionally, there were no significant differences between the two groups in terms of EQ-5D, NDI, SF-8 (physical [PCS] and mental component summaries [MCS]), JOA score, JOA recovery rate, and JOACMEQ domains. The only significant difference observed between the two groups was in the postoperative NRS score, which was significantly higher in the Group non-I (5 [3-7] vs. 2 [1-2], *p* = 0.004) (Table 3). Although 27 patients were classified as Group non-I, 17 of 27 patients had lower postoperative SPDQ scores than at baseline, and 25 of 27 patients had higher postoperative JOA scores; the JOA scores of the remaining two patients were unchanged. Thus, classification as Group non-I did not indicate an absence of symptomatic or neurological improvement after surgery.

**Table 3:** Postoperative Clinical Outcomes, PROMs and FC values.

Variable	Group I (n = 7)	Group non-I (n = 27)	*p*-value
**NRS, median [IQR]**	**2 [1-2]**	**5 [3-7]**	**0.004**^a^
EQ-5D	0.73 ± 0.22	0.77 ± 0.16	0.67^b^
NDI	7.3 ± 5.8	9.4 ± 4.8	0.40^b^
SF-8			
PCS	44.6 ± 8.7	41.6 ± 7.3	0.18^a^
MCS	50.3 ± 6.6	48.1 ± 6.7	0.50^a^
JOA score	12.7 ± 2.8	13.3 ± 1.4	0.60^b^
JOA recovery rate (%)	33.7 ± 28.3	38.8 ± 20.8	0.67^b^
JOACMEQ			
Cervical spine function	62.1 ± 46.5	64.6 ± 28.4	0.91^a^
Upper limb function	77.6 ± 25.2	81.7 ± 15.6	1.0^a^
Lower limb function	60.7 ± 38.8	66.3 ± 24.4	0.85^a^
Bladder function	80.3 ± 18.0	75.0 ± 20.4	0.65^a^
QOL	58.4 ± 20.2	50.9 ± 17.5	0.39^b^
FC1 values	−0.02 ± 0.11	−0.04 ± 0.15	0.99^c^
FC2 values	0.11 ± 0.18	0.17 ± 0.18	1.0^c^

Continuous variables are shown as mean ± SD or median [IQR], as appropriate. Statistical significance was set at *p* < 0.05.

Abbreviations: PROMs, Patient Reported Outcome Measures; NRS, Numerical Rating Scale; EQ-5D, EuroQol 5 Dimensions, 5-Level version (EQ-5D-5L); NDI, Neck Disability Index; SF-8, Short Form-8 Health Survey; PCS, Physical Component Summary; MCS, Mental Component Summary; JOA, Japanese Orthopaedic Association; JOACMEQ, JOA Cervical Myelopathy Evaluation Questionnaire; QOL, Quality of Life; IQR, Interquartile Range.

a Mann–Whitney *U* test.

b Welch's *t*-test.

c p-values are false discovery rate-corrected for multiple comparisons.

### 3.4 Correlation analysis between changes in SPDQ and PROMs

There was a positive moderate correlation observed between ΔSPDQ and ΔNRS, and a negative moderate correlation observed between ΔSPDQ, ΔEQ-5D, ΔSF-8 (PCS, MCS), and Δupper limb function of JOACMEQ. This suggests that improvements in pain, PROMs, physical function, and mental status correlated with SPDQ improvement (Supplemental Table).

The FC1 values showed only a mild correlation with all PROMs (r < 0.3, Table 4). In contrast, preoperative FC2 values showed a moderate correlation with ΔNRS (*r* = −0.33, *p* = 0.05), ΔEQ-5D (*r* = 0.32, *p* = 0.06), and ΔJOACMEQ upper limb function (r = 0.32, *p* = 0.07), suggesting that those with preoperative higher FC2 values are likely to experience greater improvement in pain, QOL, and upper limb function postoperatively (Table 5).

**Table 4:** Correlation analysis between preoperative FC1 values and changes in PROMs from preoperative to postoperative periods.

Variable	*r*	*p*-value
NRS	−0.11	0.55
EQ-5D	0.12	0.50
NDI	−0.22	0.22
SF-8		
PCS	0.23	0.20
MCS	0.23	0.19
JOACMEQ		
Cervical spine function	0.08	0.66
Upper limb function	0.29	0.09
Lower limb function	0.17	0.34
Bladder function	0.21	0.22
QOL	0.13	0.45

All correlations were computed using Spearman's rank correlation. Statistical significance was defined as *p* < 0.05.

Abbreviations: PROMs, Patient Reported Outcome Measures; NRS, Numerical Rating Scale; EQ-5D, EuroQol 5 Dimensions, 5-Level version (EQ-5D-5L); NDI, Neck Disability Index; SF-8, Short Form-8 Health Survey; PCS, Physical Component Summary; MCS, Mental Component Summary; JOA, Japanese Orthopaedic Association; JOACMEQ, JOA Cervical Myelopathy Evaluation Questionnaire; QOL, Quality of Life.

**Table 5:** Correlation analysis between preoperative FC2 values and changes in PROMs from preoperative to postoperative periods.

Variable	r	*p*-value
NRS	−0.33	0.05
EQ-5D	0.32	0.06
NDI	−0.26	0.14
SF-8		
PCS	−0.17	0.34
MCS	0.1	0.58
JOACMEQ		
Cervical spine function	0.19	0.28
Upper limb function	0.32	0.07
Lower limb function	−0.06	0.72
Bladder function	−0.31	0.07
QOL	−0.01	0.97

All correlations were computed using Spearman's rank correlation. Statistical significance was defined as *p* < 0.05.

Abbreviations: PROMs, Patient Reported Outcome Measures; NRS, Numerical Rating Scale; EQ-5D, EuroQol 5 Dimensions, 5-Level version (EQ-5D-5L); NDI, Neck Disability Index; SF-8, Short Form-8 Health Survey; PCS, Physical Component Summary; MCS, Mental Component Summary; JOA, Japanese Orthopaedic Association; JOACMEQ, JOA Cervical Myelopathy Evaluation Questionnaire; QOL, Quality of Life.

### 3.5 Multivariate analysis

Exploratory multivariate linear regression analysis for postoperative SPDQ showed that preoperative FC1 was an independent significant predictor associated with postoperative SPDQ (β = −24.05 [95% CI, −37.94 to −10.16], *p* = 0.001), whereas there were no significant effects of preoperative FC2 value (β = 6.81 [95% CI, −7.59 to 21.21], *p* = 0.34; Table 6). There were no significant effects of age, sex, or preoperative NRS score on postoperative SPDQ score (all *p* > 0.16).

**Table 6:** Multivariable linear regression analysis for postoperative SPDQ.

Variables	β (95% CI)	*p*-value
Age (years)	−0.13 (−0.32, 0.05)	0.16
Sex (male)	0.86 (−4.27, 5.99)	0.73
Preoperative NRS	0.56 (−0.34, 1.46)	0.21
**Preoperative FC1 values**	**−24.05 (−37.94, −10.16)**	**0.001**^a^
Preoperative FC2 values	6.81 (−7.59, 21.21)	0.34

Values are presented as regression coefficients (β) with 95% confidence intervals (CI), R^2^ = 0.52.

Abbreviations: SPDQ, Spine painDETECT Questionnaire; NRS, Numerical Rating Scale.

a p < 0.05 was considered statistically significant.

## 4 Discussion

In this study, patients with DCM were stratified by NP improvement. The non-improvement group exhibited higher preoperative NRS scores, which remained significantly elevated postoperatively. The unequal group sizes reflected the categorical SPDQ-based definition and should not be interpreted as indicating a lack of overall surgical benefit in Group non-I. Beyond these clinical differences, the rs-fMRI analysis revealed the following:

(1) FC1 was significantly higher in Group I and may be an independent factor associated with postoperative SPDQ improvement.
(2) FC2 was significantly higher in Group non-I and showed a moderate correlation with NRS and EQ-5D improvement.

Although FC1 and FC2 appeared to show opposing preoperative to postoperative changes in Group I, they are anatomically distinct connections, and the present analysis was not designed to assess a direct relationship between them.

These results suggest that, in addition to conventional clinical indicators such as symptom duration and pain intensity, specific preoperative FC patterns may represent exploratory candidate biomarkers associated with postoperative NP status. Given the study design and sample size, these findings should be regarded as hypothesis-generating and require validation in larger prospective cohorts.

### 4.1 Rs-fMRI and NP in previous studies

Accumulating rs-fMRI evidence indicates that NP is associated with abnormal connectivity in pain-related networks, particularly in the thalamocortical and insular–anterior cingulate circuits (Chen et al., 2025; Nardoni et al., 2025; Zhu et al., 2023). These networks are critically involved in the sensory-discriminative and affective-emotional dimensions of chronic pain, and alterations in these dimensions have been reported in various NP conditions (Zhang et al., 2021; Zhu et al., 2023; Mandloi et al., 2023).

In contrast, rs-fMRI studies of DCM have predominantly focused on motor recovery. Duggal et al. demonstrated cortical reorganization in patients with spinal cord compression (Duggal et al., 2010), Zhou et al. linked sensorimotor cortex activity with disease severity (Zhou et al., 2015), and Ryan et al. showed the longitudinal recovery of motor networks after decompression surgery (Ryan et al., 2018). Takenaka et al. provided preliminary utility of preoperative FC in postoperative motor recovery (Takenaka et al., 2019). Most prior studies described changes in sensorimotor or visual networks following decompression, but provided little direct evidence linking functional connectivity to NP outcomes.

Recent studies extended this line of research beyond motor recovery. Eto et al. reported postoperative changes in resting-state FC that paralleled the recovery in clinical scores, suggesting that abnormal preoperative connectivity may normalize after surgery. Another study using voxel-wise amplitude of low-frequency fluctuations (ALFF) reported that increased ALFF in the mid-cingulate cortex predicted postoperative axial pain, suggesting that localized spontaneous activity in this region may underlie heightened pain hypersensitivity (Su et al., 2023). Our study provides novel evidence that specific preoperative FC patterns are associated with postoperative NP prognosis in patients with DCM. This finding extends the role of rs-fMRI beyond motor networks in pain outcomes, suggesting that preoperative brain imaging may be useful in identifying patients at risk of persistent NP.

### 4.2 Significance of FC1

The finding that FC1 connectivity was significantly higher in Group I suggests a functional role of the thalamo–prefrontal network. The thalamus serves as a critical hub for pain signal relay, while the middle frontal gyrus, a part of the prefrontal cortex, is involved in the cognitive processing of pain (Baliki et al., 2012). Previous studies have reported reduced thalamic connectivity in patients with NP after brachial plexus avulsion (Zhu et al., 2023) and decreased thalamotor cortex connectivity in postoperative cervical myelopathy (Akimoto et al., 2024). These findings underscore the clinical relevance of thalamic pathways in chronic pain. In line with these observations, converging neuroimaging and interventional data suggest that the prefrontal cortex is involved in top–down modulation of thalamic and subcortical nociceptive processing through mechanisms such as attention, expectancy, and reappraisal (Urien and Wang, 2019). Within this framework, stronger thalamic–prefrontal connectivity may represent a greater capacity for the cognitive modulation of pain, effectively representing a form of resilience against maladaptive pain amplification. Furthermore, this may also help interpret the weak correlation between preoperative FC1 and NRS improvement. Patients with higher FC1 already exhibit greater cognitive modulation at baseline, leaving less room for postoperative pain improvement. In contrast, improvement in patients without FC1 resilience may be more variable due to the reduced capacity for modulation. Higher FC1 was an independent factor associated with postoperative SPDQ scores in the multivariate regression analysis. This suggests a crucial role of FC1 in postoperative NP outcomes, supporting its potential as a candidate biomarker.

### 4.3 Significance of FC2

The occipital fusiform gyrus is involved in visual information processing, and previous studies have demonstrated an altered ALFF in the visual cortex of patients with cervical myelopathy compared with healthy controls (Chen et al., 2018), as well as the predictive value of visual cortex–frontal connectivity for neurological recovery (Takenaka et al., 2019). Recovery of visual cortex connectivity postoperatively has also been reported (Chen et al., 2020), suggesting that plasticity within the visual networks may accompany functional improvements. In our cohort, the FC2 value was higher in patients without NP improvement; however, a greater preoperative FC2 correlated with larger improvements in NRS and EQ-5D scores. This may indicate that stronger engagement in visual association networks is related to the perceptual and attentional aspects of pain and body representation. This could be modified after decompression, and thereby linked to a broader improvement in pain and QOL. Although the neuropathic components of pain captured by the SPDQ remained refractory, patients still experienced improvements in overall pain perception, QOL, and upper limb function. In contrast, such a dissociation was not observed for FC1, raising the possibility that visual networks play a unique role in shaping multidimensional pain outcomes. These findings suggest that visual cortical circuits serve as modifiable substrates that influence multidimensional recovery.

### 4.4 Potential of FC as a biomarker

FC values derived from rs-fMRI can serve as predictors of treatment outcomes, such as antidepressant response in psychiatric disorders (Nemati et al., 2020), therapeutic response in diabetic NP (Teh et al., 2023), and even placebo responsiveness in chronic pain trials (Tetreault et al., 2016). These findings indicate the potential utility of FC values as biomarkers for outcome prediction, and methodological guidelines further emphasize the importance of validation and reproducibility in such predictive modeling (Scheinost et al., 2019). Although the present study was exploratory, the accumulation of more cases may allow the establishment of FC-based prediction models for postoperative NP. Such models could ultimately contribute to personalized medicine by enabling the preoperative identification of patients at higher risk of persistent pain, thereby supporting tailored perioperative management and more informed surgical counseling.

This study had some limitations. First, this was a single-center retrospective observational analysis of prospectively collected data, which limits the generalizability of our findings and precludes causal inferences. Enrollment was limited to patients who could complete the preoperative research procedures, which may have introduced selection bias. Second, the modest sample size and group imbalance may limit the statistical power and stability of the regression estimates, and bootstrap or robust sensitivity analyses were not performed. Potential confounding factors such as symptom duration (which differed between groups), surgical variables (approach, levels, operative time, and blood loss), perioperative medications, and psychological factors (e.g., anxiety, depression, catastrophizing) were not fully modeled. Third, patient classification was based on the NP status at 6 months postoperatively. In addition, the non-improvement group included both patients with persistent NP and those who developed postoperative NP despite the absence of preoperative NP. Therefore, some degree of heterogeneity in baseline NP status may have been present between groups, which should be considered when interpreting the observed FC differences. Follow-up was limited to 6 months, and clinical and rs-fMRI data at 12 and 24 months were not available. Longer longitudinal follow-up is needed to determine whether the observed NP and FC patterns persist or evolve over time. Fourth, this study identified FC alterations associated with NP improvement in an exploratory manner. Threshold determination and external validation are necessary before clinical application as a predictive model. Fifth, resting-state FC is derived from temporal correlations in BOLD signals and does not directly measure neuronal activity or cerebral oxygen metabolism; regional differences in neurovascular coupling may therefore affect FC estimates (Epp et al., 2026). In addition, because no healthy control group was included, we could not determine whether the observed FC patterns were specific to DCM or within the range of normal interindividual variation. Sixth, postoperative cervical MRI was not routinely obtained; therefore, the relationship between residual NP and postoperative structural imaging findings, including the adequacy of decompression, could not be systematically assessed. Future multicenter prospective studies with large datasets may enhance the prognostic utility and clinical translation of rs-fMRI for NP in patients with DCM.

## 5 Conclusions

In this study, preoperative rs-fMRI identified specific FC patterns associated with postoperative NP status in patients with DCM. Enhanced preoperative FC1 was associated with postoperative NP improvement. Additionally, greater FC2 was correlated with improvements in pain perception and QOL, although it did not independently predict postoperative NP status. These findings suggest that preoperative FC may provide exploratory neuroimaging correlates of postoperative NP; however, further validation is required before clinical application.

## Author contributions

TN, EF, TA, KI and MK conceived and designed the study. TN, EF, KI, YO, KS, TS (Takahiro Sunami), TS (Tomoaki Shimizu), SO, HG, TA, KM, HN, TF, MK, MY and HT gathered and analyzed the data for the study. TN and EF drafted the manuscript which was then significantly revised by TA, MK and HT. All authors approved the current version of the manuscript submitted for publication.

## Ethical approval

The institutional review board (IRB) of University of Tsukuba Hospital (approval No. H30-248) approved all procedures, including the review of patient records. All procedures were performed in accordance with the Declaration of Helsinki. All patients provided written informed consent. The IRB approved the procedures outlined for obtaining consent for this study.

## Consent

Written informed consent was obtained from all participants for the publication of this study.

## Data availability

The datasets generated and analyzed during the current study can be obtained from the corresponding author upon reasonable request.

## Declaration of generative AI and AI-assisted technologies in the manuscript preparation process

During the preparation of this work, the authors used ChatGPT (OpenAI, San Francisco, CA, USA) for language refinement and organization of the manuscript draft. After using this tool, the authors reviewed and edited the content as needed and take full responsibility for the content of the article.

## Funding

The authors did not receive support from any organization for the submitted work.

## Declaration of interest statement

The authors declare that they have no known competing financial interests or personal relationships that could have appeared to influence the work reported in this paper.
