# Supplementary material for: Preoperative functional connectivity and postoperative neuropathic pain in degenerative cervical myelopathy: An exploratory postoperative follow-up study using resting-state functional MRI
Source: Brain Spine. 2026 Aug 29;6:106661. doi: 10.1016/j.bas.2026.106661 (PMC13553560; doi:10.1016/j.bas.2026.106661)
Supplement: Supplementary file 1 [file mmc1.docx]

**Supplemental Table. Correlation between changes in SPDQ and changes in PROMs from pre- to postoperative periods.**

| **Variable** | ***r*** | ***p*-value** |
| --- | --- | --- |
| **NRS** | **0.34** | **0.047** |
| **EQ-5D** | **-0.41** | **0.015** |
| NDI | 0.27 | 0.13 |
| SF-8 |  |  |
| **PCS** | **-0.36** | **0.039** |
| **MCS** | **-0.35** | **0.043** |
| JOACMEQ |  |  |
| Cervical spine function | -0.25 | 0.16 |
| **Upper limb function** | **-0.39** | **0.024** |
| Lower limb function | -0.32 | 0.06 |
| Bladder function | -0.07 | 0.72 |
| QOL | -0.28 | 0.1 |

All correlations were computed using Spearman’s rank correlation. Statistical significance was defined as *p* < 0.05

Abbreviations: SPDQ, Spine painDETECT Questionnaire; PROMs, Patient Related Outcome Measures; NRS, Numerical Rating Scale; EQ-5D, EuroQol 5 Dimensions, 5-Level version (EQ-5D-5L); NDI, Neck Disability Index; SF-8, Short Form-8 Health Survey; PCS, Physical Component Summary; MCS, Mental Component Summary; JOA, Japanese Orthopaedic Association; JOACMEQ, JOA Cervical Myelopathy Evaluation Questionnaire; QOL, Quality of Life.
